# A multi-cohort study of the hippocampal radiomics model and its associated biological changes in Alzheimer’s Disease

**DOI:** 10.1038/s41398-024-02836-9

**Published:** 2024-02-23

**Authors:** Huwei Xia, Xiaoqian Luan, Zhengkai Bao, Qinxin Zhu, Caiyun Wen, Meihao Wang, Weihong Song

**Affiliations:** 1 Center for Geriatric Medicine and Institute of Aging, Key Laboratory of Alzheimer’s Disease of Zhejiang Province, Zhejiang Provincial Clinical Research for Mental Disorders, Wenzhou Medical University, Wenzhou, Zhejiang 325035 China; 2 Oujiang Laboratory (Zhejiang Lab for Regenerative Medicine, Vision and Brain Health), Wenzhou, Zhejiang 325000 China; 3 Department of Radiology, The First Affiliated Hospital of Wenzhou Medical University, Wenzhou, Zhejiang 325035 China

**Keywords:** Diseases, Diagnostic markers

## Abstract

There have been no previous reports of hippocampal radiomics features associated with biological functions in Alzheimer’s Disease (AD). This study aims to develop and validate a hippocampal radiomics model from structural magnetic resonance imaging (MRI) data for identifying patients with AD, and to explore the mechanism underlying the developed radiomics model using peripheral blood gene expression. In this retrospective multi-study, a radiomics model was developed based on the radiomics discovery group (*n* = 420) and validated in other cohorts. The biological functions underlying the model were identified in the radiogenomic analysis group using paired MRI and peripheral blood transcriptome analyses (*n* = 266). Mediation analysis and external validation were applied to further validate the key module and hub genes. A 12 radiomics features-based prediction model was constructed and this model showed highly robust predictive power for identifying AD patients in the validation and other three cohorts. Using radiogenomics mapping, myeloid leukocyte and neutrophil activation were enriched, and six hub genes were identified from the key module, which showed the highest correlation with the radiomics model. The correlation between hub genes and cognitive ability was confirmed using the external validation set of the AddneuroMed dataset. Mediation analysis revealed that the hippocampal radiomics model mediated the association between blood gene expression and cognitive ability. The hippocampal radiomics model can accurately identify patients with AD, while the predictive radiomics model may be driven by neutrophil-related biological pathways.

## Introduction

Alzheimer’s disease (AD) is a progressive age-related neurodegenerative disease characterized by cognitive functional impairment and is the leading cause of dementia. The mechanism underlying the development of AD is not clear, and there is currently no cure or disease-modified treatment to slow the progression of this disorder [1]. As the disease progresses, neurodegenerations produce brain atrophy, and hippocampal atrophy has been one of the most robust markers of AD [2, 3]. Structural magnetic resonance imaging (MRI) can reliably visualize anatomic pathology. However, hippocampal atrophy and/or shape changes are only a rough representation of the complex anatomical changes that occur in AD. Some pathological and microstructural changes already existed prior to overt atrophy, such as amyloid deposition, tau phosphorylation, and metabolic changes [4, 5]. Therefore, novel methods and techniques that can detect more subtle changes in the hippocampus are urgently needed.

Radiomics, a texture analysis method, can be used to extract numerous quantitative features from imaging data. Its great potential in improving diagnostic, prognostic accuracy and treatment outcomes has been increasingly explored [6, 7]. In AD, hippocampal radiomics features have been validated in classifying AD and normal controls (NC) in previous studies [8–10]. However, these studies did not evaluate the relationship between radiomics features and their underlying biological functions. The biological meaning underlying radiomics features remain poorly understood, posing a major obstacle to its broad application. In the emerging field of radiogenomics combining radiomics with genomics, early evidence has revealed the underlying cellular and molecular bases of radiomics features in brain tumor, breast, and lung cancers [11–14]. However, there have been no previous reports of hippocampal radiomics features associated with biological functions in AD.

AD is primarily viewed as a brain disorder. Many systemic manifestations have suggested that the interaction between the brain and periphery might play a functional role in AD pathogenesis and development [15, 16]. A recent meta-analysis using three public datasets found that AD-related gene expression changes in the blood are useful for early disease diagnosis and could predict AD classification [17]. Also, blood-based gene expression changes have shown similar molecular pathways for capturing neurodegenerative disease progression as identified in brain-based expression for AD [18]. Therefore, blood-based profiles provide an efficient tool for assessing the complex interplay between the brain and periphery in AD pathogenesis. Furthermore, compared to brain tissue and cerebrospinal fluid (CSF), blood is easily available, less invasive, and more amenable to large-scale screening. Therefore, it is important to explore the association among blood-based profiles, hippocampal radiomics features as well as cognitive impairment.

Our study hypothesized that dysregulation of biological pathways in the blood could be associated with altered hippocampal radiomics features, which further leads to cognitive decline in patients. In this study, we first developed a radiomics model to distinguish AD patients from controls based on hippocampal radiomics features. Our study found that the model can robustly identify AD patients. Then we further elucidated the gene expression patterns and key biological functions underlying individual radiomics features and associated cognitive ability. Our results showed that the predictive radiomics features may be driven by the activation of myeloid leukocyte and neutrophil.

## Materials and methods

### Study design

**Fig. 1:**
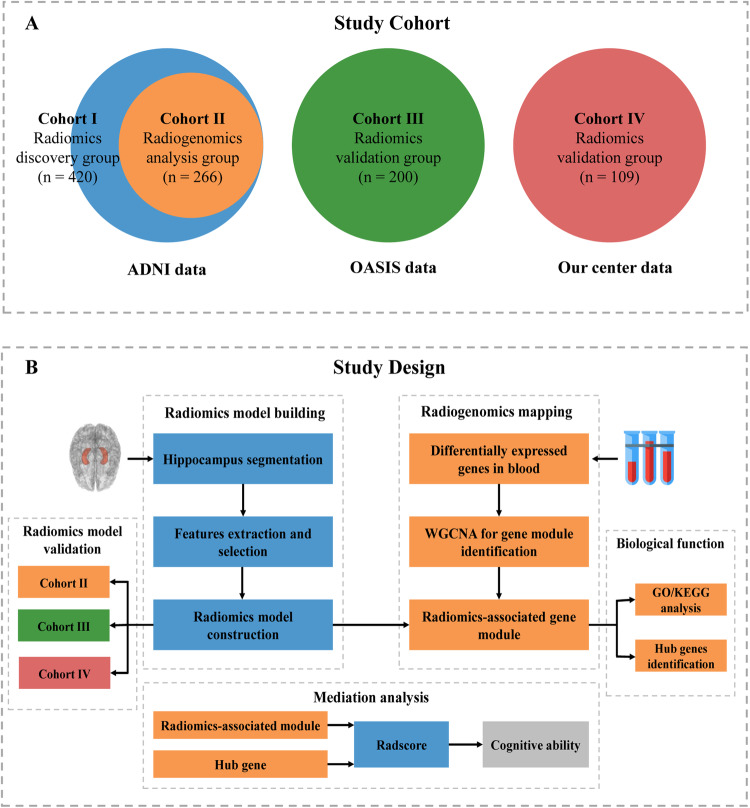
Overview of the study design. **A** Venn diagram of the four study cohorts. The study cohort consisted of 729 patients, divided into three groups: the radiomics discovery group (*n* = 420), the radiogenomics analysis group (*n* = 266, a subset of the radiomics discovery group), and the radiomics validation group (*n* = 309) from two different centers. **B** Analytical framework of the integrative radiogenomics study. Initially, a deep learning-based segmentation model was employed to automatically delineate the hippocampus. Subsequently, we extracted and chose the most meaningful predictive radiomics features to construct a radiomics signature, which enabled the differentiation of AD patients from normal controls. In addition, we conducted weighted gene co-expression network analysis (WGCNA) to identify the gene module most closely associated with radiomics and elucidated its biological role. Finally, we utilized mediation analysis to investigate the potential mediating effect of hippocampal radiomics features on the link between blood-based gene expression and cognitive ability.

A flowchart summarizing the three main steps of the study cohorts and design is illustrated in Fig. 1. First, the hippocampus was automatically delineated using a deep learning-based segmentation model. We then extracted and selected the most significant predictive radiomics features to develop a radiomics signature for identifying AD patients. Second, we identified the gene module most relevant to radiomics using weighted gene co-expression network analysis (WGCNA) and revealed its biological function. Finally, we used mediation analysis to explore whether hippocampal radiomics features mediate the relationship between blood-based gene expression and cognitive ability.

### Study population

For this retrospective multi-study, we obtained data from sources: the Alzheimer’s Disease Neuroimaging Initiative (ADNI) database, Open Access Series of Imaging Studies (OASIS) database [19], and our medical center between July 2020 and January 2022. ADNI and OASIS are open public databases, so informed consent and ethical approval were not required for access to data from these databases. Institutional review board approval was obtained at our center, and informed consent was not required due to the retrospective study design. All patients with AD from the three cohorts met the NINCDS-ADRDA criteria for probable AD after physical examination and neuropsychological testing [20]. In ADNI and OASIS, the inclusion of AD and NC participants was based on their respective diagnostic files, and detailed inclusion criteria can be found on their websites. At our center, NC participants were without reported cognitive decline complaints, and their Mini-Mental State Examination (MMSE) ≥24. Participants with a current or past history of mental and neurological disorders, including major depression, stroke, brain tumor, head injury, or cerebrovascular injury associated with cognitive impairment, were excluded from the study. As shown in Fig. 1, a total of 729 patients were in four study cohorts: Cohort I, 420 patients, consisting of two parts: patients with both blood transcriptome and 3T structural MRI in ADNI GO, and all patients with 3T structural MRI in ADNI 2. This was a radiomics discovery group used to build a radiomics model; Cohort II, 266 patients with both blood transcriptome and 3T structural MRI in ADNI GO and ADNI 2. This cohort was used as a radiogenomics analysis group to examine the biological significance of radiomics features; Cohort III, 200 patients with 3T structural MRI were randomly selected from the OASIS database. This cohort was used to validate the radiomics model; and Cohort IV, 109 patients with 3T structural MRI from our clinical center were further used to validate the radiomics model.

### MRI acquisition

All MRI scans from our center were obtained using the Philips Achieva Tx 3.0 T scanner. The parameters of the T1 structural sequence were: repetition time = 25 ms; echo time = 46 ms; matrix = 256 × 256 × 155; and voxel resolution = 1.0 mm × 1.0 mm × 1.0 mm. The details of the ADNI and OASIS MRI data acquisition can be found on the ADNI website (http://adni.loni.usc.edu/) and the OASIS website (http://www.oasis-brains.org/). The quality of the images was assessed for lack of patient motion and distortion artefacts.

### Hippocampus segmentation and radiomics features extraction

To minimize the bias caused by segmentation uncertainty and increase effectiveness, the automated deep learning-based hippocampal segmentation toolkit Hippodeep was used to segment the bilateral hippocampi [21]. Previous studies have shown that this tool has good reliability and internal consistency for hippocampal segmentation [22, 23].

Based on the segmentation results, radiomics features extracted from both hippocampi were used to quantify the tissue spatial heterogeneity. All images were first corrected for the bias field using the N4 algorithm [24] and resampled to obtain an isotropic image of 1 × 1 × 1 mm^3^. Subsequently, 107 radiomics features (summarized in Supplementary Table 1) characterizing the hippocampus shape, intensity, and texture patterns were extracted from each hippocampal mask. In total, 214 (107 * bilateral hippocampus) radiomics features were obtained. An open-source Python package PyRadiomics (version 3.0.1; https://pyradiomics.readthedocs.io/) was used to extract a standardized set of radiomics features [25].

### Radiomics model construction and validation

The radiomics discovery group (Cohort I) was randomly divided into training (*n* = 295) and validation (*n* = 125) groups at a ratio of 7:3, with balanced patient characteristics. To establish a radiomics prediction model in the training group, we used two feature selection methods, the minimum redundancy maximum relevance (mRMR) and least absolute shrinkage and selection operator (LASSO) algorithms, to select the most discriminative features and avoid overfitting. mRMR was performed first to remove redundant and irrelevant features, and the top 30 features with the highest correlation were selected. LASSO regression was executed to select the final features for training. A tenfold cross-validation was performed to determine the optimal parameters based on deviance. This process can compress the coefficients of some features to zero, and nonzero coefficient features were finally retained to build a radiomics score (Radscore) using multivariate logistic regression analysis to identify patients with AD. The violin plots were plotted to show the differences in Radscore across group. The area under the curve (AUC) values using receiver operating characteristic (ROC) analysis were used to evaluate the prediction performance of the Radscore among the cohorts.

### Gene co-expression modules construction and radiogenomics analysis

Gene expression data were obtained from blood samples of Cohort II in the ADNI. More details about sample collection and pre-processing of gene expression data can be found on the ADNI website. We used a processed gene expression profile that contained 48,157 genes. Duplicate probes of genes were aggregated by computing the median values and then isolating the protein-coding genes. Finally, the gene expression data of 16,728 genes were obtained. Differential gene expression analysis was performed using the “limma” package in R software [26]. Genes with *P* value <0.05 were considered as differentially expressed genes (DEGs) between the AD and NC groups. To explore the correlation between DEGs and radiomics features, the R package “WGCNA” was used to construct a gene co-expression network and identify highly correlated gene modules [27]. Each module was represented by the module eigengene (ME). A Pearson correlation coefficient was calculated to determine the relationship between MEs and radiomics features, which were visualized using a radiogenomics map. Finally, the module with the highest correlation with Radscore was considered the key module for subsequent analysis.

### Identification of biological changes and hub genes underlying radiomics features

To determine the potential biological changes associated with the radiomics features, Gene Ontology (GO) enrichment analysis and Kyoto Encyclopedia of Genes and Genomes (KEGG) pathway analysis were performed within the radiomics-associated module using the R package “clusterProfiler” [28]. Enrichment terms less than the FDR threshold of 0.05 were regarded as significant enrichment. Enriched terms were used to biologically annotate the radiomics features. Hub genes were defined as genes with high intramodular connectivity in the key module. Gene significance (GS) was determined using the Pearson correlation between module genes and radiomics features, along with module membership (MM), which was determined using the correlation between gene expression and MEs. Genes with GS >0.2 and MM >0.8 were considered the hub genes in the key module for further study.

### External validation of hub genes in the AddneuroMed dataset

To further confirm the role of these hub genes in AD progression, we downloaded a public database of AddNeuroMed dataset ANM1 from GSE63060 [29]. The ANM consortium is an independent multi-cohort European study focusing on AD biomarker discovery [30]. Blood transcriptome samples from ANM1 were analysed using the Illumina HumanHT-12 V3.0 expression BeadChip, and the detailed data processing was described in a previous paper [31]. A total of 218 subjects (130 ADs, 88 NCs) with both blood hub gene expression and cognitive ability assessed by MMSE score were used for external validation. Spearman correlation was used to analyse the correlation between hub genes expression and MMSE scores.

### Statistical analysis

For patient characteristics, categorical variables were compared using the *χ*^2^ or Fisher exact test when appropriate. Continuous data were analysed with the Student’s *t*-test or the Mann–Whitney *U*-test. Missing data were imputed using the expectation-maximization method. Results with two-tailed *p* < 0.05 were considered statistically significant. Mediation analyses were conducted using Model 4 of the PROCESS v4.0 macro developed by Andrew F. The bootstrap method with 5000 samples was used to estimate the coefficients of indirect effects. The mediation analysis comprised total, direct, and indirect effects and we used age, sex, and education as covariates. Patient characteristics and mediation analyses were performed using SPSS (version 25.0). Radiomics feature extraction was performed using Python (version 3.7.15) and the rest of the analysis was performed using R studio (version 4.1.2).

## Results

### Patient characteristics

Of the 729 patients enrolled in this study, 420 were in the radiomics discovery group (Cohort I), 266 were in the radiogenomics analysis group (Cohort II, a subgroup of the radiomics discovery group), and 309 were in the radiomics validation group from two centers(Cohort III and Cohort IV), as shown in Fig. 1. The demographic and clinical information of Cohort I is summarized in Tables 1, 2. Demographic and clinical information for the remaining cohorts is provided in Table 3.

**Table 1:** Characteristics of patients in the training and test sets.

Variable	Training set (*n* = 295)	Test set (*n* = 125)	*p* value
Age, year	75.42 (7.396)	74.96 (7.114)	0.559
Sex, male	145 (49.15%)	68 (54.4%)	0.325
Education, year	16.06 (2.780)	16.10 (2.668)	0.905
APOE4	134 (45.42%)	45 (36%)	0.074
ADAS-Cog13	17.51 (12.220)	19.42 (13.977)	0.186
MMSE	26.61 (3.554)	26.32 (3.732)	0.461

**Table 2:** Clinical characteristics of NC and AD patients in radiomics discovery group.

Variable	Training set (*n* = 295)			Test set (*n* = 125)		
	NC (*n* = 177)	AD (*n* = 118)	*p* value	NC (*n* = 75)	AD (*n* = 50)	*p* value
Age, year	75.60 (6.927)	75.14 (8.072)	0.595	74.76 (6.358)	75.26 (8.176)	0.702
Sex, male	76 (42.94%)	69 (58.47%)	0.009	39 (52%)	29 (58%)	0.509
Education, year	16.25 (2.717)	15.78 (2.862)	0.156	16.61 (2.493)	15.32 (2.759)	0.007
APOE4	50 (28.25%)	84 (71.19%)	0.000	19 (25.33%)	26 (52%)	0.002
ADAS-Cog13	8.97 (4.462)	30.32 (8.336)	0.000	9.46 (4.42)	34.35 (9.25)	0.000
MMSE	28.95 (1.296)	23.09 (2.903)	0.000	29.08 (1.112)	22.18 (2.057)	0.000

**Table 3:** Clinical characteristics of NC and AD patients in Cohort II-IV.

Variable	Cohort II (*n* = 266)			Cohort III (*n* = 200)			Cohort IV (*n* = 109)		
	NC (*n* = 206)	AD (*n* = 60)	*p* value	NC (*n* = 100)	AD (*n* = 100)	*p* value	NC (*n* = 63)	AD (*n* = 46)	*p* value
Age, year	75.90 (6.618)	76.63 (8.727)	0.484	68.18 (7.642)	75.52 (7.176)	0.000	64.56 (6.455)	71.09 (8.503)	0.000
Sex, male	92 (44.66%)	36 (60%)	0.036	37 (37%)	55 (55%)	0.011	23 (36.5%)	22 (47.8%)	0.236
Education, year	16.40 (2.681)	15.32 (3.061)	0.008	15.56 (2.560)	14.94 (3.061)	0.122	**/**	**/**	**/**
APOE4	58 (28.16%)	43 (71.67%)	0.000	37 (37%)	64 (64%)	0.000	**/**	**/**	**/**
ADAS-Cog13	9.01 (4.04)	31.96 (9.11)	0.000	**/**	**/**	**/**	**/**	**/**	**/**
MMSE	29.00 (1.20)	22.17 (3.40)	0.000	28.95 (1.321)	24.61 (3.440)	0.000	26.86 (5.047)	17.97 (8.597)	0.000

### Radiomics model construction and validation

**Fig. 2:**
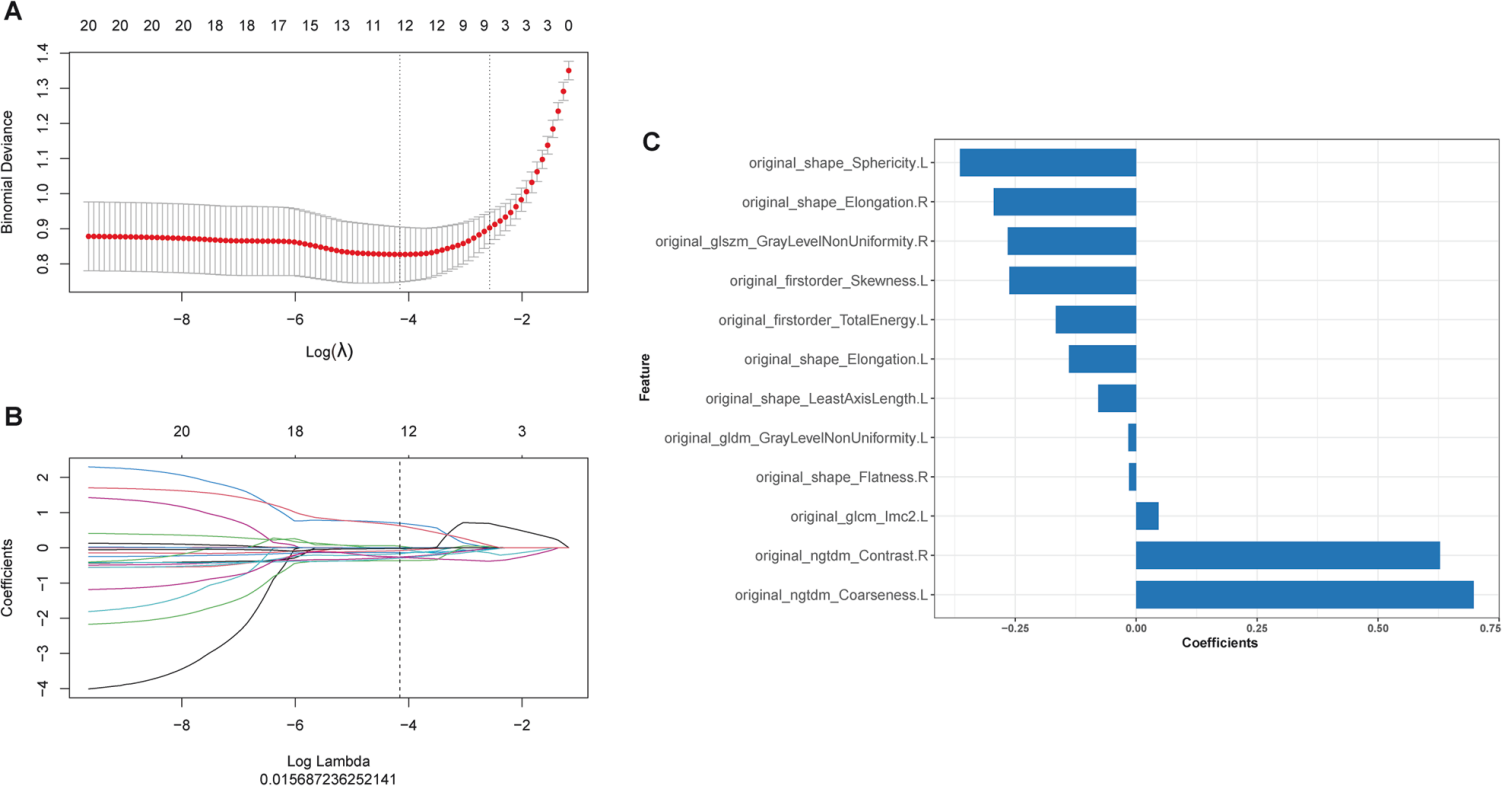
A summary of feature selection results. **A** Selection of the optimal hyperparameter parameter (λ) in the LASSO model via tenfold cross-validation based on minimum criteria. Binomial deviances from the LASSO regression cross-validation procedure were plotted as a function of log (λ). The optimal λ value of 0.016 was selected. **B** The black vertical line was drawn at the value selected using tenfold cross-validation in **A**. The 12 resulting features with nonzero coefficients were indicated in the plot. **C** The remaining 12 features after feature selection, the y-axis represents each radiomics features and the x-axis represents their coefficients.

Twelve features remained to establish the Radscore. Using a lasso logistic regression model (λ = 0.016), the histogram of the Radscore is shown in Fig. 2, and the final formula of the Radscore was as follows:

Radscore = −0.139*original_shape_Elongation.L-0.016*original_gldm_GrayLevelNonUniformity.L-0.262*original_firstorder_Skewness.L-0.364*original_shape_Sphericity.L + 0.698*original_ngtdm_Coarseness.L-0.294*original_shape_Elongation.R-0.078*original_shape_LeastAxisLength.L + 0.046*original_glcm_Imc2.L-0.166*original_firstorder_TotalEnergy.L-0.266*original_glszm_GrayLevelNonUniformity.R-0.015*original_shape_Flatness.R + 0.628*original_ngtdm_Contrast.R -0.471.

All Radscore of patients with AD were significantly higher than those of NC patients in the four cohorts, and the results are displayed as a violin plot in Supplementary Fig. 1. The AUC of the radiomics model was 0.91 (95% confidence interval [CI], 0.88–0.95) in the training cohort and 0.91 (95% CI, 0.86–0.97) in the validation cohort. In the other three cohorts, the radiomics model also showed strong discriminative power, with AUC values of 0.93 (95% CI, 0.89–0.97), 0.91 (95% CI, 0.87–0.95), and 0.87 (95% CI, 0.80–0.95), respectively (Supplementary Fig. 2).

### Gene co-expression module construction and radiogenomics analysis

**Fig. 3:**
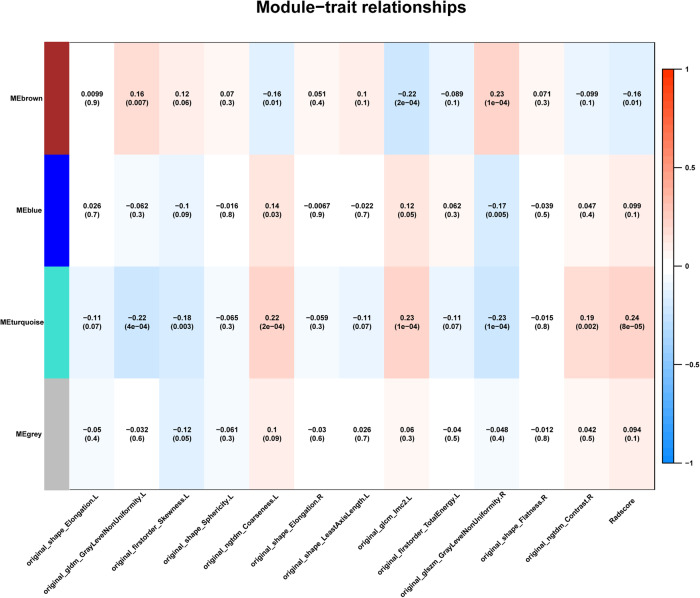
Radiogenomics map of paired correlation between gene modules and radiomics features used in calculating the Radscore. Gene modules were generated based on gene expression similarity of differentially expressed genes between the AD and NC groups. The turquoise module exhibited the strongest correlation with Radscore, as shown by the highest correlation coefficient (*r* = 0.24, *p* = 8e-5) in the map.

We chose an appropriate soft threshold power β = 4 to build a scale-free co-expression network, the scale-free *R*^2^ was 0.85 and the slope was −0.98. Four gene modules were derived based on the similarity of the expression levels between genes. A radiogenomics map of the association between the selected radiomics features and gene modules is shown in Fig. 3. According to the map, the turquoise module showed the highest correlation with the Radscore (*r* = 0.24, *p* = 8e-5) and was selected for further biological analysis.

### Biological meaning and hub genes underlying radiomics features

**Fig. 4:**
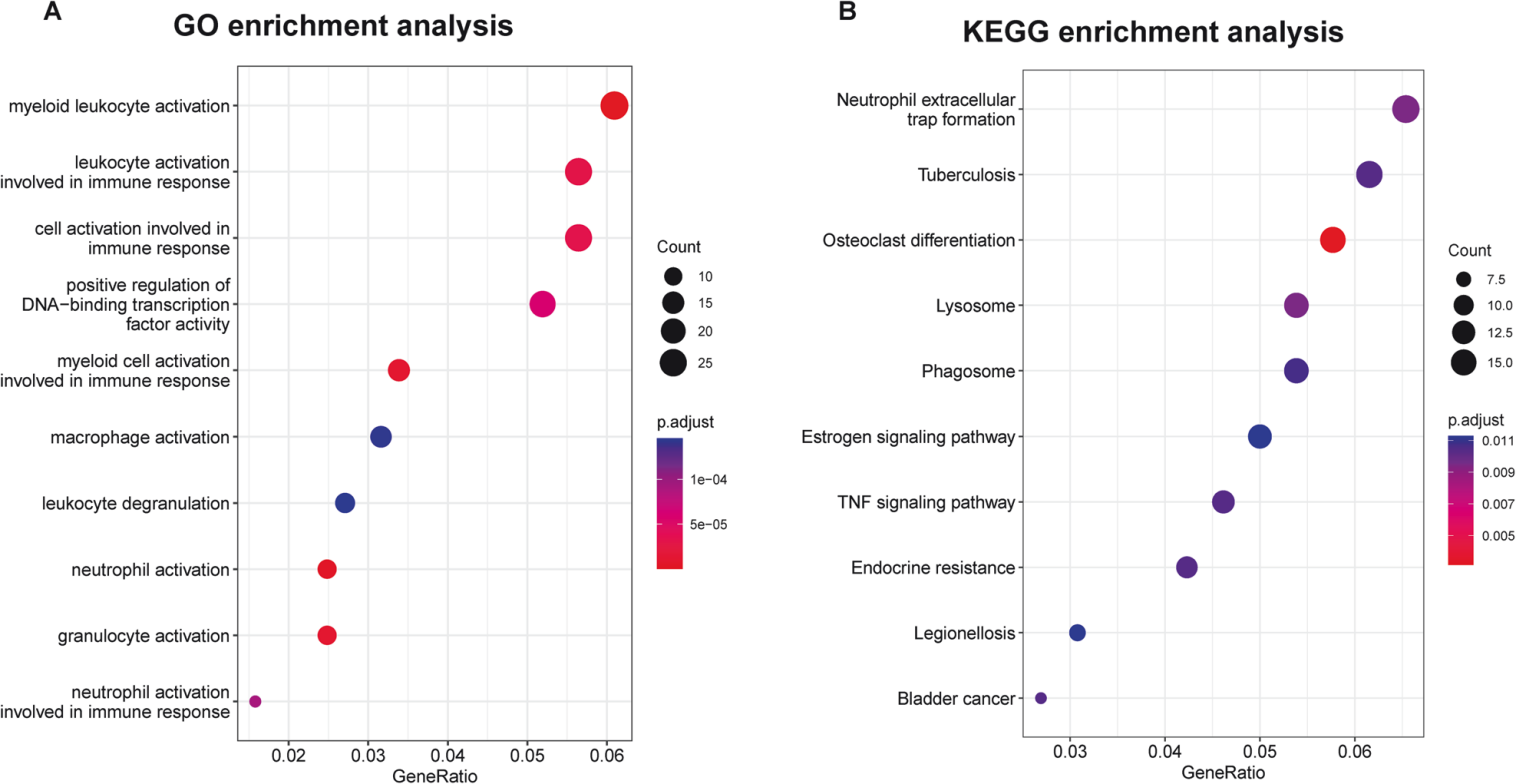
Functional enrichment analysis of the turquoise module that was significantly associated with the Radscore. **A** GO enrichment analysis revealed that the genes within the key module exhibited enrichment in myeloid leukocyte activation and neutrophil activation. **B** KEGG enrichment analysis identified significant enrichment in the neutrophil extracellular trap formation pathway.

We performed GO and KEGG analyses to reveal potential biological functions in the key module of turquoise, and the enrichment results are shown in Fig. 4. The results showed that genes in the key module were enriched in myeloid leukocyte activation and neutrophil activation, which could prove to be biological processes involved in AD development. Furthermore, KEGG enrichment analysis results indicated that neutrophil extracellular trap formation was significantly enriched. Under the thresholds of GS >0.2 and MM >0.8, we identified the following six hub genes in the key module: TMBIM6, IL1R2, LTB4R, MMP9, PADI4, and PRKCD.

### External validation of hub genes in the AddneuroMed dataset

**Fig. 5:**
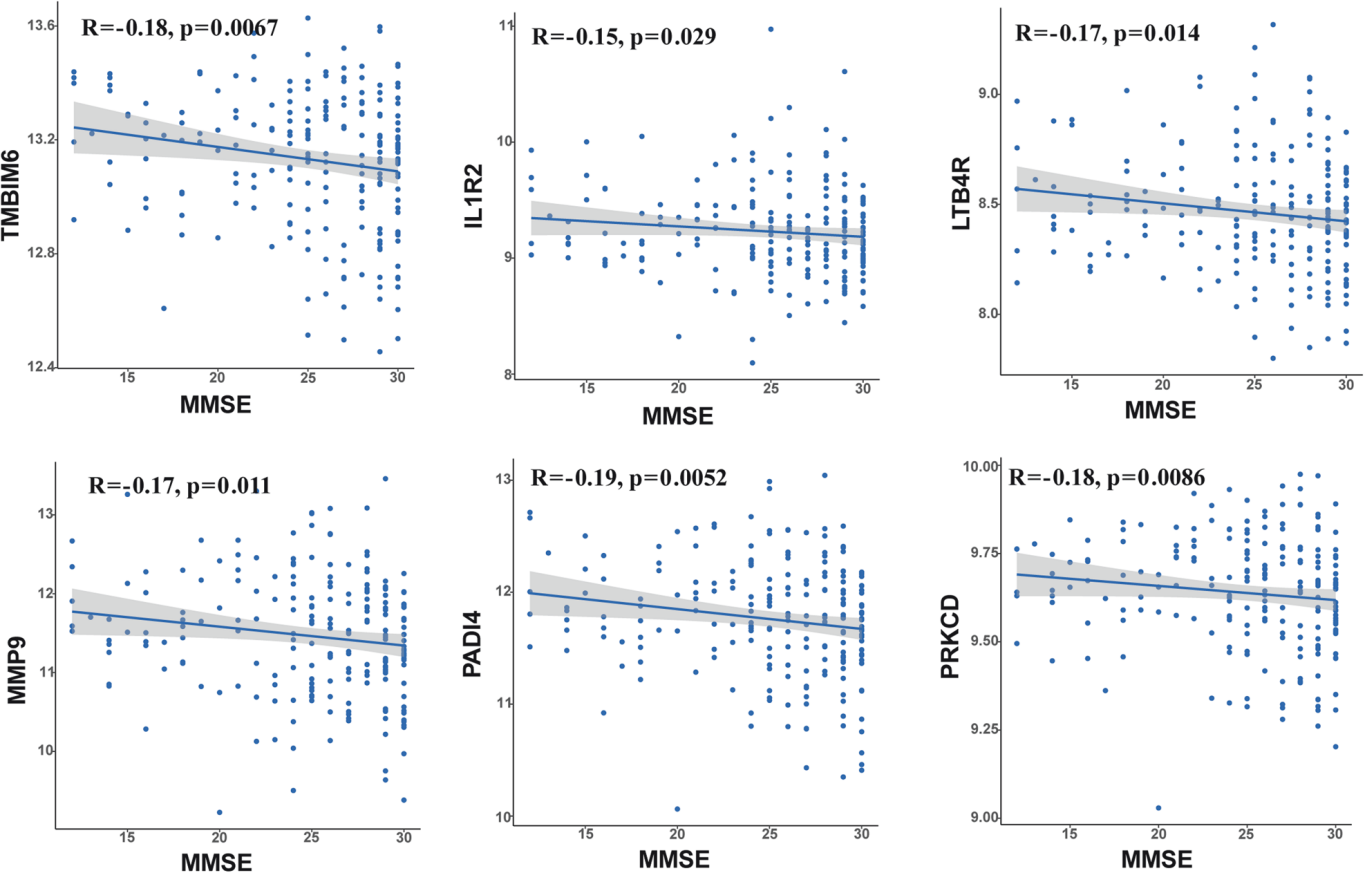
Scatter plot of correlation between mRNA expression of the six hub genes and MMSE score in ANM1. The expression of each hub gene exhibited a significant negative correlation with MMSE scores (TMBIM6: *R* = −0.18, *p* = 0.0067; IL1R2: *R* = −0.15, *p* = 0.029; LTB4R: *R* = −0.17, *p* = 0.014; MMP9: *R* = −0.17, *p* = 0.011; PADI4: *R* = −0.19, *p* = 0.0052; PRKCD: *R* = −0.18, *p* = 0.0086).

The results are shown in a scatter plot (Fig. 5) with Spearman correlation coefficients (R), and the related *p* values are given. As expected, each hub gene expression negatively correlated with MMSE score (TMBIM6: *r* = −0.18, *p* = 0.0067; IL1R2: *r* = −0.15, *p* = 0.029; LTB4R: *r* = −0.17, *p* = 0.014; MMP9: *r* = −0.17, *p* = 0.011; PADI4: *r* = −0.19, *p* = 0.0052; PRKCD: *r* = −0.18, *p* = 0.0086).

### Mediation analysis of blood gene expression, radiomics features, and cognitive ability

**Fig. 6:**
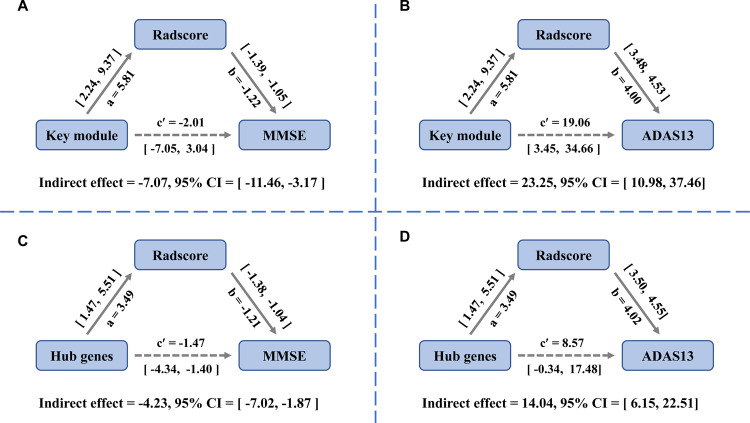
Mediation analysis for the role of radiomics features in the association between gene expression and cognitive ability. **A** Mediation analysis demonstrated a significant indirect effect of Radscore for key module gene expression and MMSE (indirect effect = −7.07, 95% CI −11.46 to −3.17). **B** Mediation analysis demonstrated a significant indirect effect of Radscore for key module gene expression and ADAS 13 (indirect effect = 23.25, 95% CI 10.98–37.46). **C** Mediation analysis demonstrated a significant indirect effect of Radscore for hub genes expression and MMSE (indirect effect = −4.23, 95% CI −7.02 to −1.87). **D** Mediation analysis demonstrated a significant indirect effect of Radscore for hub genes expression and ADAS 13 (indirect effect = 14.04, 95% CI 6.15 to 22.51).

Cognitive ability was evaluated using the MMSE and Alzheimer’s Disease Assessment Scale-cognitive subscale 13 (ADAS-Cog13). In the mediation analysis, the hippocampal radiomics features showed significant mediation effects between the gene expression of the key module and cognitive ability after correcting for multiple covariates (MMSE: indirect effect = −7.07, 95% CI −11.46–−3.17; ADAS 13: indirect effect = 23.25, 95% CI 10.98–37.46; Fig. 6). Next, we explored whether hub genes played an equally important role in the mediation analysis. The results also showed that the expression of hub genes mediated the hippocampal radiomics features, which in turn led to cognitive function changes (MMSE: indirect effect = −4.23, 95% CI −7.02–−1.87; ADAS 13: indirect effect = 14.04, 95% CI 6.15–22.51; Fig. 6).

## Discussion

In this multicenter study, we constructed and validated an MRI-based hippocampal radiomics model for predicting patients with AD, and further revealed the key biological functions underlying hippocampal radiomics. Finally, mediation analysis was used to explore the relationship between gene expression, radiomics features, and cognitive ability. The main findings of our study were: (1) The Radscore calculated based on the selected 12 hippocampal radiomics features showed good performance in predicting AD patients with excellent stability and reproducibility. (2) Myeloid leukocyte and neutrophil activation were significantly associated with hippocampal radiomics features through radiogenomics mapping. (3) Six hub genes were selected according to each gene’s modular connectivity and radiomics relationships in the key module. Moreover, the relationship between hub genes and cognitive ability was validated using an external validation set of ANM1. (4) Revealed the hippocampal radiomics features can serve as an intermediate phenotype to elucidate the relationship between blood gene expression and cognitive ability.

Neuroimaging plays a vital role in the diagnosis and monitoring of AD progression. MRI can reflect brain tissue structure intuitively and noninvasively, has become a primary tool in neuroscience and research on neurodegenerative diseases [32–35]. In addition to hippocampal atrophy, abnormal energy metabolism, functional connectivity disruption, and microstructural changes in the hippocampus also have been well-reported [36–40]. Given the complexity of the hippocampus in AD, we used an MRI-based radiomics model to differentiate between patients with AD and NC. Many studies have emphasized the importance of radiomics [6], and considered it an intermediate phenotype to unravel the relationship between genetic variants and disease pathophysiology [41]. Our results showed that hippocampal radiomics features could identify AD with an AUC of 0.929 in the ADNI radiogenomics subgroup, 0.907 in the OASIS group, and 0.874 in our center group. The excellent predictive performance found by our analysis is consistent with recent studies [8–10]. The advantage of our study is that we used a deep learning model to effectively segment the hippocampus with competitive accuracy. Furthermore, we conducted a multicenter validation study to confirm the stability and robustness of the model. These results together highlight the significance of MRI-based hippocampal radiomics features as a promising neuroimaging biomarker for AD. The model is simple and feasible, and supplements existing classical markers with a wide range of potential applications.

Notably, the underlying biological meaning of radiomics remains a scientific challenge that should be clarified before its translation into clinical practice. In this study, we constructed a weighted gene co-expression module for the remaining genes following the identification of blood gene expression alterations in AD. We then evaluated the association between the gene modules and radiomics features. The results showed that the turquoise module was most related to Radscore, and the genes of this module were mainly enriched in neutrophil-related biological pathways. Neutrophils, as the most abundant leukocyte type in the peripheral blood, can migrate efficiently to sites of infection to eliminate invading pathogens [42]. Currently, complex and adaptable functions of neutrophils in acute injury and repair, cancer, and chronic inflammatory processes are increasingly being recognized [43]. Although the role of neutrophils in AD development has not been fully elucidated, it has been proposed that the neutrophil phenotype could be related to the rate of cognitive decline [44]. Moreover, the results of animal experiments showed that neutrophils could release inflammatory mediators and intravascular neutrophil extracellular traps, thus inducing blood-brain barrier disruption and cognitive deficits [45]. In another study, brain capillaries were blocked by neutrophils, leading to decreased cerebral blood flow in an AD mouse model [46]. All these studies support our results that radiomics features are closely related to neutrophil-related biological functions. To further explore the relationship between these variables, we conducted a mediation analysis. Based on our results, we can reasonably speculate that peripheral blood neutrophils may impair the hippocampus, leading to cognitive decline in AD patients. The details of this mechanism should be elucidated in future studies. Our findings add to the growing body of research highlighting the importance of neutrophils in AD development, thus potentially providing a new direction for therapy.

We also selected six hub genes in the key module: TMBIM6, IL1R2, LTB4R, MMP9, PADI4, and PRKCD. Of these hub genes, TMBIM6 was significantly dysregulated in AD in a blood-based transcriptome-wide meta-analysis [47]. Moreover, this gene was a binding protein of free presenilin 1, the catalytic subunit of γ-secretase responsible for the rate-limiting step in producing Aβ [48]. An association between the IL1R2 rs34043159 and AD has been identified among the Chinese population [49]. And in AD mouse models, inhibition of BACE1 in microglia can enhance the phagocytosis of microglia by inhibiting IL1R2 and Toll-like receptors, correlated with a significant reduction in Aβ [50]. LTBR4 is an important target for reducing inflammation. Although there is no direct evidence of the correlation between LTBR4 and AD pathogenesis, it can be detected as abnormally elevated in neurons and glial cells in postmortem brain tissues of patients with AD [51]. MMP9 is an endopeptidase that is involved in angiogenesis and inflammation. In both mice and humans, this gene is known to be associated with cognitive ability and may act as a potential marker for AD [52–54]. PADI4 was found accumulated in the hippocampus and cerebral cortex of patients with AD and may contribute to the formation of autoantibodies [55]. PRKCD is known to be related to neurodegeneration induced by Aβ [56]. Similarly, our results showed the expression of hub genes found to be significantly associated with cognitive ability in the external validation data. In addition, the results of the mediation analysis also showed that hub gene expression is related to cognitive ability through the mediation of radiomics features. Above all, these results support a strong association between hub genes and AD. However, few studies focus on the mechanisms of AD-related pathological processes, the biological mechanism needs to be urgently explored. With further in-depth research, these genes could be used as possible therapeutic targets or diagnostic markers.

To our knowledge, this is the first report to explore the underlying biological interpretation of hippocampal radiomic features in a multicenter population-based cohort study. Nevertheless, it is inevitable that our research still had some limitations. First, this was a retrospective study, and thus biases and confounding factors are unavoidable, although we used a relatively large sample size and the samples were from multiple centers. Second, our radiogenomic mapping findings were hypothesis-generating, although mediation analysis and external validation were conducted, further animal experiments are required to verify the exact underlying mechanisms.

## Conclusions

We constructed a hippocampal radiomics model with good performance for the noninvasive identification of patients with AD. The underlying biological meaning of the radiomics model is the neutrophil-related biological pathway. In addition, these radiomics features could serve as a critical intermediate phenotype used to illuminate the intricate linkages between peripheral blood gene expression and cognitive ability. These results shed light on the biological interpretation of the radiomics model and provide new insights into the treatment and prevention of AD.

### Supplementary information

Supplementary Information

## Data Availability

All data generated or analysed during this study are included in this published article, and the authors agree the availability upon request.
