# Supplementary material for: A multi-cohort study of the hippocampal radiomics model and its associated biological changes in Alzheimer’s Disease
Source: Transl Psychiatry. 2024 Feb 23;14:111. doi: 10.1038/s41398-024-02836-9 (PMC10891125; doi:10.1038/s41398-024-02836-9)
Supplement: Supplementary file 1 — Supplementary Information [file 41398_2024_2836_MOESM1_ESM.docx]

**Supplementary Information**

**Supplementary Fig 1.** Violin distribution of the Radscore of NC and AD patients in the four study cohorts.

**Supplementary Fig 2.** ROC curves of the Radscore in identifying AD patients.

**Supplementary Table 1.** Radiomics features extracted from Pyradiomics.

**Figure legends**

**Supplementary Fig 1.** Violin distribution of the Radscore of NC and AD patients in the four study cohorts. The figure showed that the all Radscore values of AD patients were significantly higher than those of NC patients in each subgroup.

**Supplementary Fig 2:** ROC curves of the Radscore in identifying AD patients. **A:** ROC curve on the Radiomics discovery group. In the training and validation cohorts, the radiomics model demonstrated AUCs of 0.91 (95% CI, 0.88-0.95) and 0.91 (95% CI, 0.86-0.97), respectively. **B:** ROC curve on the cohort Ⅱ-Ⅳ. In the cohort Ⅱ-Ⅳ, the radiomics model demonstrated AUCs of 0.93 (95% CI, 0.89-0.97), 0.91 (95% CI, 0.87-0.95) and 0.87 (95% CI, 0.80-0.95), respectively.

**Figures**


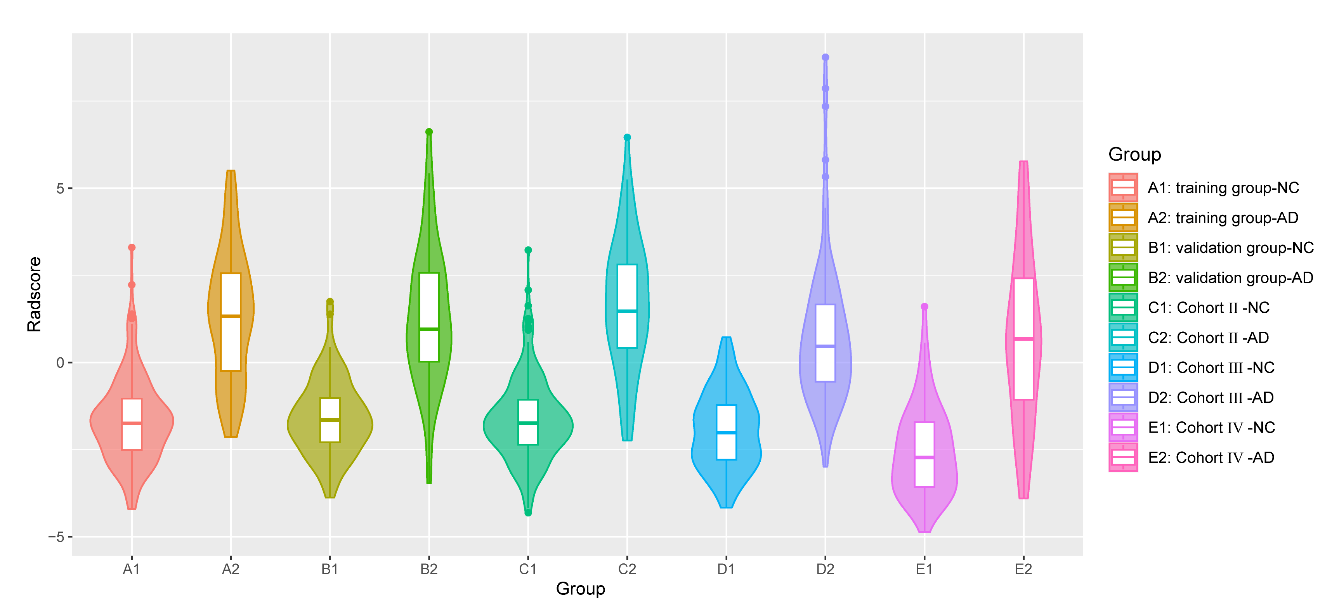


Supplementary Fig 1


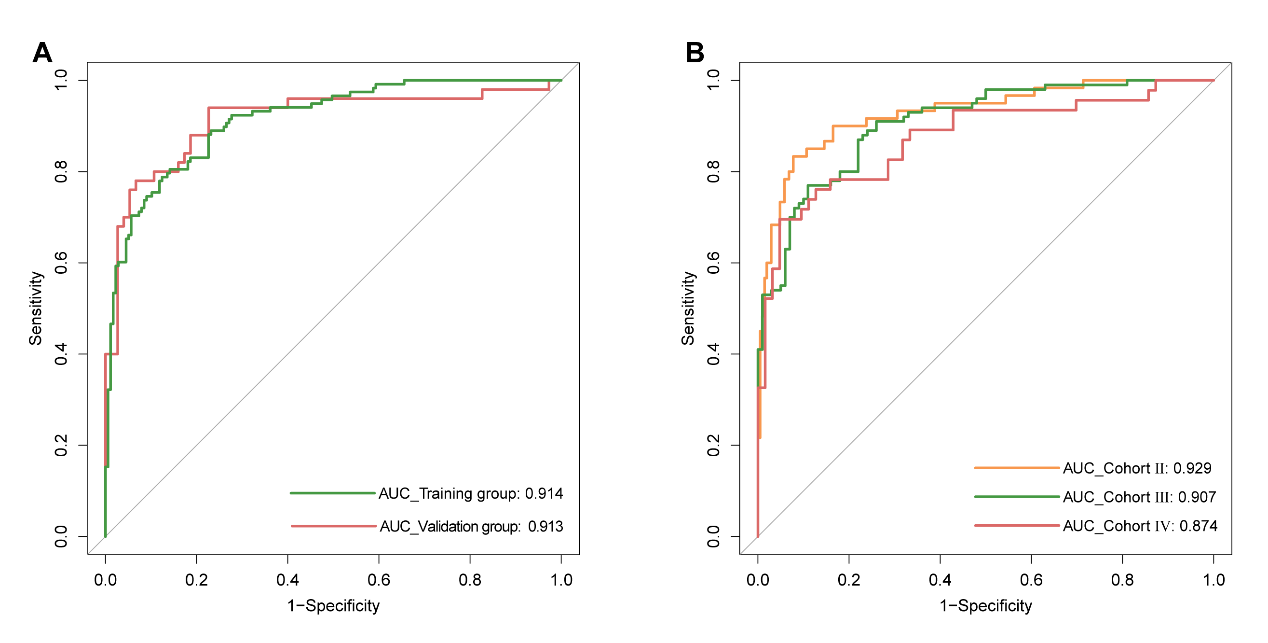


Supplementary Fig 2

**Supplementary Table 1. Radiomics features extracted from Pyradiomics**

| Feature category | Feature list |
| --- | --- |
| Shape (n=14) | Volume, surface area, surface area to volume ratio, sphericity, maximum 3D diameter, maximum 2D diameter (column), maximum 2D diameter (row), maximum 2D diameter (slice), major axis, minor axis, least axis, elongation, flatness, mesh volume |
| First-order statistics (n=18) | Energy, total energy, entropy, minimum, 10^th^ percentile, 90^th^ percentile, maximum, mean, median, interquartile range, range, mean absolute deviation, robust mean absolute deviation, root mean squared, skewness, kurtosis, variance, uniformity |
| GLCM (n=24) | Autocorrelation, cluster prominence, cluster shade, cluster tendency, contrast, correlation, difference average, difference entropy, difference variance, inverse difference, inverse difference moment, inverse difference moment normalized, inverse difference normalized, informal measure of correlation 1, informal measure of correlation 2, inverse variance, joint average, joint energy, joint entropy, maximal correlation coefficient, maximum probability, sum average, sum entropy, sum of squares |
| GLRLM (n=16) | Short-run emphasis, long-run emphasis, gray level nonuniformity, gray level nonuniformity normalized, run-length nonuniformity, run-length nonuniformity normalized, run percentage, gray level variance, run variance, run entropy, low gray level run emphasis, high gray level run emphasis, short-run low gray level emphasis, short-run high gray level emphasis, long-run low gray level emphasis, long-run high gray level emphasis |
| GLSZM (n=16) | Small area emphasis, large area emphasis, gray level non-uniformity, gray level non-uniformity normalized, size-zone non-uniformity, size-zone non-uniformity normalized, zone percentage, gray level variance, zone variance, zone entropy, low gray level zone emphasis, high gray level zone emphasis, small area low gray level emphasis, small area high gray level emphasis, large area low gray level emphasis, large area high gray level emphasis |
| GLDM (n=14) | Small dependence emphasis, large dependence emphasis, gray level non-uniformity, dependence non-uniformity, dependence non-uniformity normalized, gray level variance, dependence variance, dependence entropy, low gray level emphasis, high gray level emphasis, small dependence low gray level emphasis, small dependence high gray level emphasis, large dependence low gray level emphasis, large dependence high gray level emphasis, |
| NTGDM (n=5) | Coarseness, complexity, strength, contrast, busyness |

GLCM = gray level co-occurrence matrix, GLDM = gray level dependence matrix, GLRLM = gray level run-length matrix, GLSZM = gray level size zone matrix, NTGDM = neighboring gray tone difference matrix.
